# Droplets Formation and Merging in Two-Phase Flow Microfluidics

**DOI:** 10.3390/ijms12042572

**Published:** 2011-04-15

**Authors:** Hao Gu, Michel H. G. Duits, Frieder Mugele

**Affiliations:** Physics of Complex Fluids, Faculty of Science and Technology, IMPACT and MESA + Institutes, University of Twente, P.O. Box 217, 7500AE Enschede, The Netherlands; E-Mails: m.h.g.duits@utwente.nl (M.H.G.D.); f.mugele@utwente.nl (F.M.)

**Keywords:** microfluidics, two-phase flow, droplet formation, droplet merging, electro-coalescence, electrowetting

## Abstract

Two-phase flow microfluidics is emerging as a popular technology for a wide range of applications involving high throughput such as encapsulation, chemical synthesis and biochemical assays. Within this platform, the formation and merging of droplets inside an immiscible carrier fluid are two key procedures: (i) the emulsification step should lead to a very well controlled drop size (distribution); and (ii) the use of droplet as micro-reactors requires a reliable merging. A novel trend within this field is the use of additional active means of control besides the commonly used hydrodynamic manipulation. Electric fields are especially suitable for this, due to quantitative control over the amplitude and time dependence of the signals, and the flexibility in designing micro-electrode geometries. With this, the formation and merging of droplets can be achieved on-demand and with high precision. In this review on two-phase flow microfluidics, particular emphasis is given on these aspects. Also recent innovations in microfabrication technologies used for this purpose will be discussed.

## 1. Introduction

Nowadays, microfluidics can be found in numerous applications, such as emulsification, chemical synthesis, biomedical diagnostics and drug screening [1–4]. Compared to conventional techniques that use reaction vessels, test tubes or microtiter plates, microfluidic technology offers several unique advantages: (i) much less volume of sample or reagents is used, which is practical and reduces costs; (ii) the diagnostic results or the molecular products are obtained in a shorter time, because the high surface-to-volume ratios at the microscale lead to shorter heat and mass transfer times; (iii) miniaturization allows for an increase in parallelization and automation. For instance it offers a way of screening and systematic testing in the domain of drug discovery.

In the initial development of microfluidics in the 1990s, mostly continuous flow systems were considered. These systems were more or less derived from macroscopic setups, with the principal aim to reduce reagent consumption. The desire to further downscale the amounts of reagents and to reduce the processing time have remained as a driving force ever since. However inherently, in the processing of single phase liquids in continuous flow, molecular reagents or products can become distributed over the entire liquid which fills the channel. This effect, known as Taylor-Aris dispersion [5] gives rise to lower concentrations, with possible adverse consequences for the efficiency of chemical reactions, or the detection of (molecular) species.

As an alternative platform, droplet-based microfluidics has also been developed. In this approach, all molecular processes are confined to the volume of a single drop, allowing for even stronger reductions in reagent volume and reaction time. A second advantage of using droplets is that the contact with solid walls is eliminated. This strongly reduces problems due to adsorption of dissolved components to the channel walls, and increases the efficiency of chemical reactions. And thirdly, new functionalities can be implemented: simple Boolean logic functions can be performed in droplet microfluidic systems [6–8].

One of the commonly used platforms for droplet-based microfluidics is based on devices with closed microchannels. Discrete droplets are then produced in a continuously flowing immiscible liquid, and manipulated by downstream changes in the flow, either passively via e.g., bifurcations or constrictions, or actively using e.g., valves or electric fields. This platform, being a subset of two-phase flow (TPF) microfluidics, offers unique possibilities for producing droplets with sizes in the nanometer to micrometer range in a controlled and reproducible manner, also with a high throughput. The generated droplets can subsequently be used in several types of lab-on-a-chip applications, for example as microvessels for chemical or biochemical reactions, to be initiated by merging two droplets. Generally, droplet-based TPF is best suited to continuous processes like the production of emulsions or the encapsulation of a large number of biological targets [9–11].

Effective utilization of the possibilities offered by the droplet-based TPF platform, also requires tuning the chemistry and physics of the device (and its operation) to that of the application. As a first example, surfactants play an important role. They reduce the interfacial tension between the dispersed and continuous phases, thereby facilitating surface deformation (as is needed during the formation of droplets), flow through constrictions or droplet splitting. Generally, surfactants also stabilize drops against coalescence. By residing at the interface of the two fluid phases with their hydrophilic heads in the aqueous phase and hydrophobic tails in the oil phase, surfactants can turn unstable emulsion droplets into metastable colloids. A direct consequence of this stabilization is that it also becomes more difficult to let two such droplets merge when this is needed.

A second example where chemistry enters the picture concerns the wettability of the inner surfaces of the microfluidic chip. In droplet-based TPF, the continuous phase should wet the channel surface favorably, whereas the dispersed phase should be disfavored by the channel walls. For instance, aqueous droplets suspended in oil need hydrophobic channels, whereas oil-in-water emulsions require hydrophilic channels. Hence the materials used for fabricating microchannels and surface modification technologies are quite important for producing and manipulating droplets.

Physics comes into play when considering the formation of droplets, their flow behavior and when they need to be manipulated via external fields. The manipulation of droplets with high precision and flexibility is still an important issue. In particular the generation of droplets *on demand* or merging them at certain location still poses challenges in many cases. Different approaches for droplet formation, merging, splitting and sorting are currently explored by many research groups. Besides hydrodynamic manipulation, also electrical control is increasingly used in microfluidic devices, especially for generating and merging droplets. While this contribution of physics is not limited to channel flow geometries, but can also be found in planar geometries (so called digital microfluidics using embedded electrode patterns to actuate the drops), we will in the current review restrict ourselves to the former case. The latter case has been reviewed in [12–14].

This review is further organized as follows: First, we will briefly introduce the physical parameters which are considered in a fluidic system. Subsequently, we will review the state-of-the-art in droplet formation and droplet merging under both the hydrodynamic and electric control conditions. Finally, microfluidic device consideration for various applications will be discussed.

## 2. Dimensionless Numbers

In engineering, the behavior of liquids is often described in terms of dimensionless numbers which compare the importance of different physical properties. The Bond number *Bo* = Δ*ρgL*^2^/*σ*, with Δ*ρ* the difference in mass density between the two fluids, *g* the gravity acceleration, *L* a characteristic length scale, and *σ* the interfacial tension, compares gravitational and surface forces [15]. In microfluidic applications, generally *Bo* << 1, this means that gravity effects can be ignored. The Reynolds number *Re* = *ρνL*/*μ*, where *ρ* is the mass density, *μ* the dynamic viscosity and *ν* the mean velocity of the fluid, compares inertial and viscous forces. Generally, in microfluidics *Re* < 1 [16]. A third quantity is the Weber number, which compares inertial forces to surface forces: *We* = *ρν*^2^*L*/*σ*. Also *We* < 1 in most applications at the microscale. From the definitions and typical magnitudes of Re and We, it follows that inertia generally becomes unimportant when the flow geometry is downscaled to dimensions in the micron range [10]. Exceptions include flows at very high speeds as they sometimes occur in flow focusing and co-flow devices [17], and the moment of the breakup of droplets. Otherwise the dominant forces at the microscale are interfacial forces and viscous forces.

The relative strength of these two is represented by the (dimensionless) Capillary number *Ca*, expressed by *Ca* = *μν*/*σ*. Here *μ* is generally the viscosity of the most viscous fluid in the two-phase system, *ν* is the velocity of that phase, and *σ* is the interfacial tension as before. Inherently, the interfacial tension tends to reduce the interfacial area, which is crucial in the formation of droplets and also for their subsequent stability. In many flow situations, viscous forces act to extend and stretch the interface [18]. At low *Ca* (<1) the interfacial tension dominates, and spherical droplets are found. In contrast, at high *Ca* (≫1) the viscous forces play an important role, leading to deformation of the droplets and sometimes to asymmetric shapes. In co-flowing liquid streams, high *Ca* numbers can induce a transition between different drop generation scenarios [19,20]. In some cases of high *Ca*, a completely different flow architecture, named stratified flow, can occur [21,22]. The latter is beyond the scope of this review.

## 3. Droplet Formation

Droplet formation can be considered as the first step in the microfluidic life cycle. Many different techniques have been developed to obtain fine control over the size (distributions) and shape of droplets [2]. Techniques for producing droplets can be either passive or active, the latter meaning that external fields are activated at the time and on-chip location where droplets need to be formed. Active methods will be considered in Section 3.3. The majority of techniques are passive and produce a continuous stream of evenly spaced drops [23]. In this scenario, the flow field causes the interface between the two fluids to deform, leading to a growth of interfacial instabilities. Besides a continuous mechanical pressure (by pressure controllers or hydrostatic heads) or displacement (by pumps), no external actuation or moving parts are used. Generally this allows production of droplet size distributions with standard deviations (*i.e.*, polydispersity) as small as 1–3%.

The two most common strategies are the use of T-junction and flow focusing geometries. In general, the fluid phase to be dispersed is brought into a microchannel by a pressure-driven flow, while the flow of the second immiscible carrier liquid is driven independently. These two phases meet at a junction, where the local flow field, determined by the geometry of the junction and the flow rates of the two fluids, deforms the interface. Eventually droplets pinch off from the dispersed phase finger by a free surface instability. The pinch-off of droplets is largely dictated by the competition between viscous shear stresses acting to deform the liquid interface and capillary pressure acting to resist the deformation, which is expressed by *Ca*. This number ranges between 10^−3^ and 10 in most microfluidic droplet formation devices. Quantitative predictions of the regimes of drop formation, and the drop size still pose a challenge, although significant progress has been made through analytical and numerical studies [24–27].

### 3.1. T-Junction Devices

**Figure 1.**
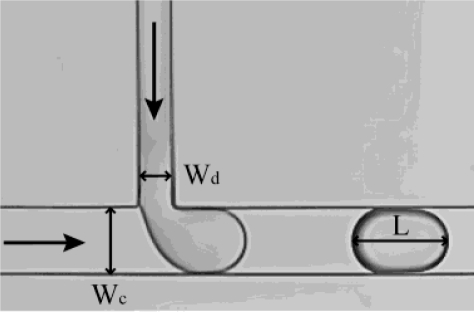
Droplet formation in a T-junction. The dispersed phase and continuous phase meet in a T-shaped junction perpendicularly. (*W_d_*: 50 μm; *W_c_*: 100 μm).

In a typical T-junction configuration, as depicted in Figure 1, the two phases flow through orthogonal channels and form droplets where they meet. This type of geometry was first demonstrated in 2001 by Thorsen *et al.* [28], who produced monodisperse droplets with pressure controlled laminar flow in microchannels. Since then many studies were performed using T-junction geometries, to achieve a better understanding of the droplet formation mechanism and the role of several physical parameters therein [25,29–35], as well as to develop various applications [36–42]. The size of the droplets depends on the flow rates of the two liquids [28], the dimensions of the channels [29], the relative viscosity between the two phases [43], and surfactants and their concentrations [44].

Three main regimes can be distinguished for drop formation as the parameters are varied: dripping, squeezing and parallel flowing stream. In the dripping regime, droplet breakup occurs when the viscous shear stress overcomes the interfacial tension, analogous to the breakup of spherical droplets. If the capillary number is chosen large enough, the droplets are emitted before they can block the channel. Alternatively if the capillary number is low, the formed droplets will obstruct the channel and hence restrict the continuous phase. This causes a dramatic increase of the hydrodynamic pressure in the upstream part, which in turn induces the pinch-off of droplets. This is the so-called squeezing regime, which has been described by Garstecki *et al.* [29]. One theoretical study about the transition from squeezing to dripping based on the influence of *Ca* and viscosity ratio was reported by Menech *et al*. [45]. Also Lattice Boltzmann simulations have been performed to increase the understanding of drop formation at T-junctions. Van der Graaf *et al.* [24] obtained a scaling rule for the drop size. Gupta *et al.* [25] found that the transition from droplet formation to parallel flows is strongly dependent on the *Ca* of the continuous phase.

**Figure 2.**
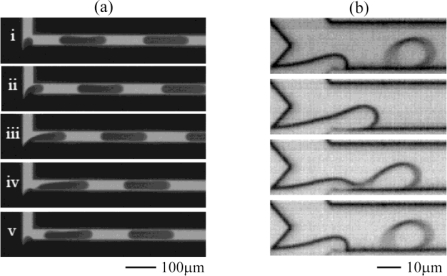
head-on devices (**a**) T-shaped junction, time sequence of droplet formation in the regime of squeezing. Reprinted from [47] with permission from University of Twente; (**b**) Y-shaped junction, time sequence of droplet formation in the dripping regime. Reprinted from [37] with permission from the American Chemical Society (ACS).

A slightly different geometry having similar features as the above explained T-junction geometry is the so-called head-on device (see Figure 2a). Shui *et al.* demonstrated droplet formation in such a device, where two liquids come from opposite directions of two straight channels and form droplets upon meeting [36,46,47]. Also a Y-shaped junction has also been studied, for example by Steegmans *et al.* [34,37]. As illustrated in Figure 2b, droplets can be formed in the dripping regime in such a Y junction geometry. The mentioned authors studied the mechanism of droplet formation and derived a general model predicting the droplet size. They also demonstrated that such a flat Y-junction can be used as a microfluidic tensiometer, *i.e.*, a device that can measure dynamic interfacial tensions.

**Figure 3.**
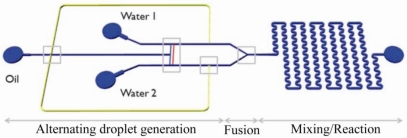
Microfluidic chip with various passive droplet manipulation capabilities. The system includes a droplet-pair generator (double T-junction), a Y-junction for droplet fusion and a winding channel for further mixing. Reprinted from [51] with permission from the Royal Society of Chemistry (RSC).

For certain applications, a single T-junction is clearly not enough. To perform chemical reactions or to produce droplets with alternating compositions, more sophisticated designs have been realized: for example double T-junctions to produce droplet pairs [48–51]. One example is shown in Figure 3 [51]. The authors of this paper demonstrated a perfect “one-to-one” droplet pair formation (self synchro-nization) with the use of additional connections in the upstream and downstream channels.

For the mass production of emulsion droplets using microfluidic devices, large scale integration of droplet generators is a necessity. For the case of T-junctions, this has been explored for up to 256 junctions in parallel [41,42]. The highest throughput was reported as 320 mL·h^−1^ in a 4 cm × 4 cm chip with 256 droplet formation units. Further developments along this line would be needed to achieve production at industrial scales, but the perspectives are already there. One of the challenges that may have to be faced is to minimize detrimental cross-talk between the different droplet injectors. This could occur for example if the transient pressure variation associated with the creation of a droplet is transmitted to other droplet injectors and interferes with the droplet formation there.

### 3.2. Flow Focusing Devices

**Figure 4.**
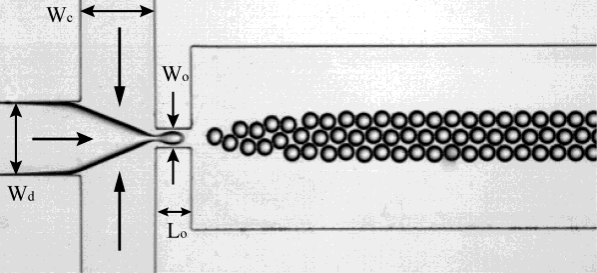
Droplet formation in flow focusing device (FFD). The widths of the inlets of dispersed phase and continuous phase, as well as the orifice are indicated as *W*_d_, *W*_c_ and *W*_o_ (*W*_d_ = *W*_c_ = 200 μm; *W*_o_ = 50 μm). The length of orifice is indicated as *L*_o_ (100 μm).

The flow focusing (FF) geometry was first proposed by Anna *et al.* [52] and Dreyfus *et al.* [53]. As demonstrated in Figure 4, it consists of three inlet channels converging into a main channel via a narrow orifice. The dispersed phase, contained in the middle channel, is squeezed by continuous phase flows from two opposing side channels. Both phases pass through the small orifice that is located downstream of the three channels. Finally, the stream of the dispersed phase becomes narrow and breaks into droplets. The droplet size is determined by the flow rates of the two phases and by the flow rate ratio [54,55], in addition to the channel geometries [56] and the viscosities of the two phases [57,58]. This multitude of influential parameters in principle offers a lot of control over drop formation, but it is also true that in the absence of adequate (*i.e.*, quantitatively predicting) theoretical models, each new combination of geometry, speeds and viscosities may need to be explored and tuned, in order to let the chip meet the demands (*i.e.*, criteria for droplet size and formation rate). Many variations of the basic flow focusing device (FFD) geometry have recently been developed to improve the control over the size and size distribution of the droplets [26,59–61]. Since recently, these developments can be assisted by numerical (Lattice Boltzmann) simulations, which have seen their first application to flow-focusing [26] and cross-flow geometries [27].

**Figure 5.**
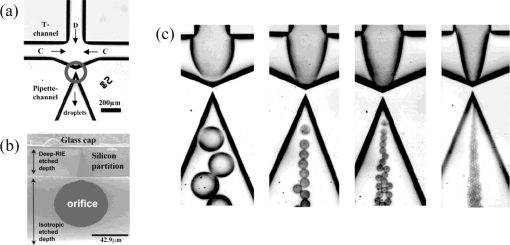
Axisymmetric flow focusing design: (**a**) planar view; (**b**) SEM image of the circular orifice; (**c**) water droplets formation at increasing oil flow rates and fixed water flow rate. Reprinted from [61] with permission from Royal Society of Chemistry (RSC).

Also so-called axisymmetric flow focusing designs have been presented (Figure 5). They allow the formation of monodisperse droplets with reduced size as compared to planar FFDs [59]. In these geometries, the dispersed phase is confined in the central axis of the microchannel, and pinches off by a combination of shear stresses and wetting upon contact with the inner surfaces of the channel.

**Figure 6.**
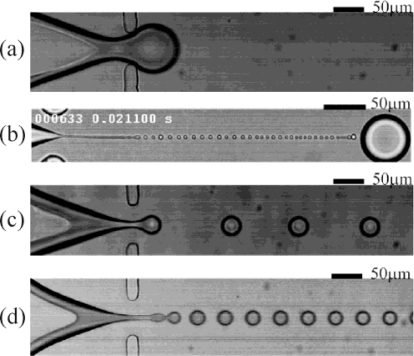
Different droplet breakup processes: (**a**) squeezing; (**b**) tip-streaming; (**c**) dripping, and (**d**) jetting. Reprinted from [56] with permission from the American Institute of Physics (AIP).

Four different droplet breakup regimes have been identified in planar FFDs: squeezing, dripping, jetting and thread formation (tip-streaming), shown in Figure 6. As mentioned, there are no general scaling laws that can predict the transitions between these regimes, and the same applies for the size and generation frequency of droplets. This is due to the large number of variable parameters. Recently, Funfschilling *et al.* concluded from velocity field measurements that the squeezing regime is governed by the build-up of a pressure difference, as a response to the partial and temporal blocking of the orifice by the advancing finger [62]. Lee *et al.* stated that the squeezing and dripping regimes depend solely on the upstream geometry and the related flow field, while the thread formation mode depends only on the downstream channel and its associated flow field [56]. It is clear that unraveling the mechanisms of droplet break-up in FFDs still needs further investigation.

Some aspects, namely the transition between dripping and jetting was studied in detail for the somewhat simpler geometry of co-flowing liquid jets [17,19,20]. Building on earlier work for liquid jets injected into an unconfined bath of another immiscible liquid [63,64], Guillot *et al.* performed a linear stability analysis of a liquid jet of the to-be-dispersed inner phase in a co-flowing stream of the outer fluid confined to a cylindrical capillary. They showed that the difference between dripping and jetting is primarily controlled by the Ca number of the outer phase fluid and be the radius of the inner jet (measured in units of the radius of the capillary). Small Ca and small radii correspond to absolutely unstable jets, *i.e.*, any perturbation of the radius propagate towards the nozzle and induce drop generation there. This corresponds to the dripping regime. For large Ca and large radii, perturbations of the radius are advected by the flow downstream along the jet where they may eventually lead to the formation of drops. In this case, the jets are said to be convectively unstable. For jet radii close to the radius of the capillary, the confinement of the inner jet plays a key role in the suppression of the break-up. Utada *et al.* [17] demonstrated yet another mechanism of dripping to jetting transition: for moderate *Ca* (<O(1)) of the outer phase jetting can be induced by increasing the flow rate of the inner phase. In this case, the We number of the inner phase was shown to be the control parameter governing the transition.

**Figure 7.**
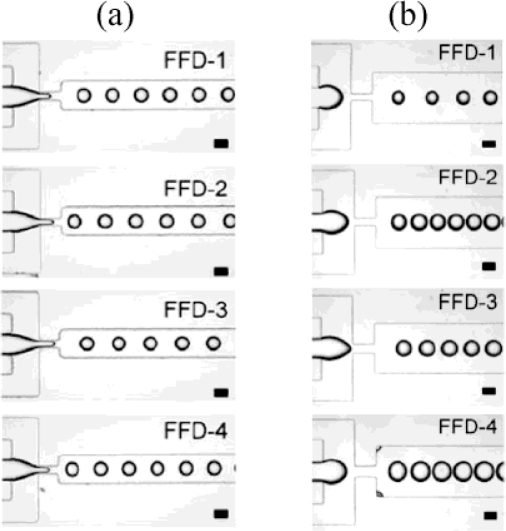
Quadruple droplet generator: (**a**) same dimensions of the orifices from FFD-1 to FFD-4; (**b**) FFDs with different widths of the orifices. Reprinted from [65] with permission from Royal Society of Chemistry (RSC).

Returning to applied aspects of drop generation, it was demonstrated that multiple FFDs could be combined in parallel in either linear [65,66] or circular circuits [41] in order to increase the droplet production rate. Li *et al.* demonstrated a quadruple droplet generator with a weak parametric coupling between the different parallel FFDs. By choosing different geometries for the individual FFDs, these authors were able to simultaneously produce several populations of droplets with distinct sizes, where each of the populations had a narrow size distribution (Figure 7). Also Hashimoto *et al.* [66] studied the dynamic mechanism of droplet formation in parallel FFDs. They found a weak hydrodynamic coupling as well.

### 3.3. Droplet Formation Assisted by Active Elements

**Figure 8.**
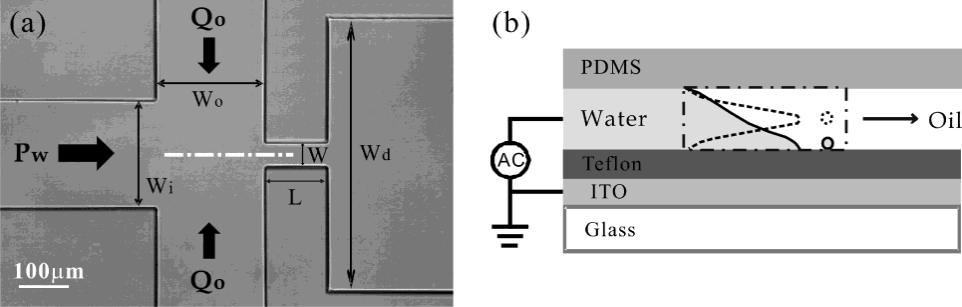
FFD with electrowetting functionality. Droplets are formed in the area indicated by dashed lines. (**a**) Top view; (**b**) Side view. An ITO layer provides the electrode while a Teflon layer provides the insulator. Activating the electrode gives rise to enhanced contact between the water finger and the bottom wall, which facilitates the formation of a droplet. Reprinted from [68] with permission from the American Institute of Physics (AIP).

**Figure 9.**
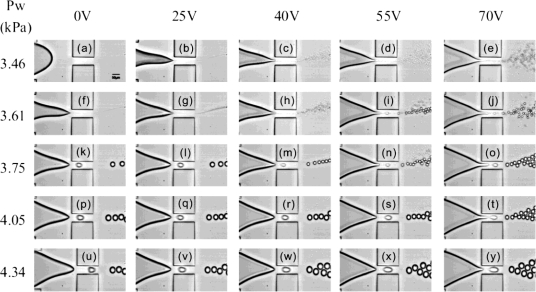
Enhanced capabilities for aqueous droplet generation by using electrowetting. Tuning besides the water flow rate also the voltage, allows obtaining a variety of droplet sizes and generation rates (scale bar is 50 μm). Reprinted from [68] with permission from the American Institute of Physics (AIP).

To increase the flexibility of FFDs, additional (active) elements have also been incorporated into devices. Several groups have applied electrical means to obtain more control over droplet formation in FFDs [67–70]. For example, Gu *et al.* integrated the functionality of electrowetting (EW) [68,69] by embedding insulator-covered electrodes underneath the channel. In this case the wetting angle between the oil/water interface with the channel wall can be tuned via the voltage applied over the insulator layer (see Figure 8). This is dictated by the so-called electrowetting number *η = ɛɛ_0_U^2^/2dσ* with *ɛɛ_0_* the permittivity and *d* the thickness of the insulator layer, *U* the voltage and *σ* the oil/water interfacial tension [71]. Three different droplet formation regimes could be achieved using the combination of hydrostatic and electrical driving: dripping, tip-streaming and conical spray (see Figure 9). It is clear from these results that the additional electric control can provide an extension of the size range in which droplets can be produced: from tens of micrometers down to a few microns in the presence of an applied voltage. Also the rate of droplet generation could be raised to very high values using EW.

The conical spray was found at high relative flow rates of the dispersed (*i.e.*, water) phase and large electrowetting numbers (*η* > 1, corresponding to *U* ≈ 50 V). In this specific regime of electro-wetting, the droplets appear to repel each other due to finite electrostatic charges accumulated at their surfaces. Similar droplet spray patterns were observed by Kim *et al.* [67] and He *et al.* [70] who integrated an electrospray functionality into FFDs. In such devices, the droplet size can also be diminished by increasing the voltage. Yet for the formation of very fine droplets, one needs to be in the Taylor cone regime, which requires voltages above ≈1500 V.

Also membrane valves have been introduced into FFDs to vary the width of the orifice [72–75]. Abate *et al.* demonstrated that the droplet size and formation frequency in the dripping regime can be controlled by such an adaptable orifice [75]. The approach based on (local) adjustment of the temperature has been reported: here use is made of the temperature dependence of the viscosity and interfacial tension [76–78]. This method allowed independently controlling the droplet size and generation frequency.

Additional means to actively control drop formation can be obtained by adding particles that respond to external fields into the to-be-dispersed phase. Examples of this approach, which we will not address in more detail here, include magnetic control of ferrofluid droplets [79], electric control of electrorheological droplets [80] and temperature control of certain types of nanofluids [81].

### 3.4. Droplet-on-Demand

In a large majority of the existing continuous flow devices, droplets are produced incessantly; the flow can be switched on and off, and the conditions of droplet generation can be modified, but the droplets will always appear in trains. In cases where this scenario is undesirable, and droplets need to become available one-by-one upon request, digital microfluidics applications [12,13] may come to mind first. However, continuous flow systems can also be adapted to deliver *droplet*-*on-demand* (DOD). Surprisingly, this possibility has hardly been explored, in spite of its strong potential for high throughput screening in microtiter technology or in the programmed coalescence of droplets (after a synchronized formation of droplet pairs). Especially the combination of *on demand* formation of droplets and a subsequent processing at high speed would make it interesting.

**Figure 10.**
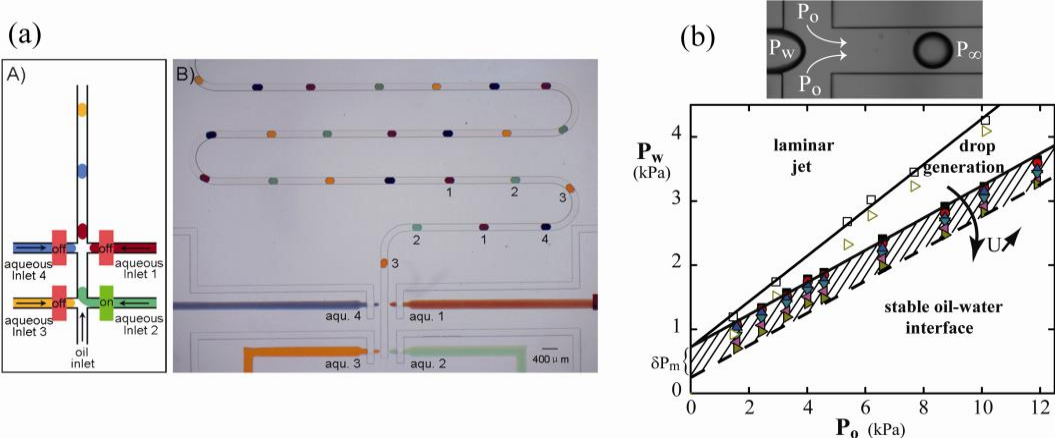
(**a**) On-demand formation of arrays of droplets with distinct composition by sequentially switching on/off microvalves. Reprinted from [82] with permission from the Royal Society of Chemistry; (**b**) Phase diagram for EW induced droplet formation. In the hatched area, droplets can be formed on demand. Reprinted from [83] with permission from IOP publishing.

One of the possibilities for on-demand droplet formation is the use of integrated microvalves [82,84,85]. For instance, Zeng *et al.* incorporated a pneumatic valve made of polydimethylosiloxane (PDMS) into microfluidic devices (Figure 10a). By intermittently switching the valve on/off, individual droplets can be produced *on demand* [82]. Also piezoelectric actuators have been used in DOD applications [86,87]. In such systems the droplet size and frequency can be set with high accuracy through a conversion of the piezo voltage into a mechanical displacement. Churski *et al.* reported a DOD system that used external electromagnetic valves interconnected with the chip for the scanning of reaction conditions [88].

Alternatively, electric fields can also be used to produce droplets on demand. Malloggi *et al.* [83,89] used electrowetting as an active control mechanism to increase the wettability of the channel wall at the location of droplet formation. Combining pressure control over the two phases and electrical control over wetting, the size and/or generation rate of their droplets could be tuned within a certain range (Figure 10b).

## 4. Droplet Merging

Droplets can be used as independent microreactors for a number of chemical and biological applications, e.g., chemical synthesis, kinetics studies, the screening of biological contents and bio-medical diagnostics. The merging of two droplets is a key step in this approach since (in the large majority of cases) this forms the trigger to start the chemical reaction(s). Practical prerequisites for merging are that the droplets (i) touch each other and (ii) overcome the stabilizing forces caused by surface tension and lubrication. Several designs have been used to bring droplets together [90–98]. To subsequently overcome the stabilizing forces, both the viscosity ratio of two-phase fluids [39], and the presence of surfactant at the interface [99–101] have to be considered.

Surfactants are generally used to stabilize emulsion droplets against coalescence. These molecules generally consist of a compact polar head and a long-chain hydrophobic tail. Surfactants reduce the interfacial tension between two liquids by adsorbing at the liquid-liquid interface where they often align perpendicular to the surface. Stabilization of droplets can be realized in different ways: (i) via repulsion between the interfaces due to electrostatic and/or steric effects; (ii) by slowing down the hydrodynamic flow along the interface via Marangoni effects or via enhanced surface viscosity [102,103]. Basically, there are two main approaches, namely passive merging and active merging, to coalesce droplets. In the case of passive merging, droplets are normally not stabilized by surfactant. Then coalescence occurs spontaneously when the droplets meet; the occurrence of which can be organized with a suitably shaped channel geometry [91]. For droplets that are stabilized by surfactants, active merging is required. For this, thermocapillary effect [76,104] or electrocoalescence [105–110] can be used.

### 4.1. Passive Merging

In passive droplet merging, the design of the channel geometry is a key to achieve proper merging, since droplet synchronization is required and active means to compensate for any synchronization errors are missing. In principle merging can occur simply at a channel junction, if the generation and transport of each pair of droplets is such that both drops arrive there at the same time. However in practice this can be difficult to achieve, and therefore special designs of geometries are often used.

**Figure 11.**
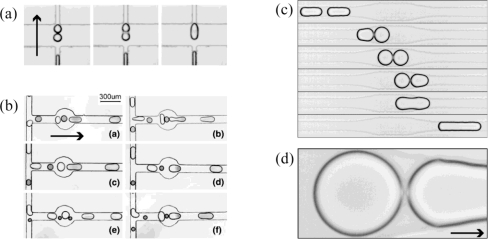
Passive droplet merging realized by design of the channel geometry. (**a**) and (**b**) show the merging of two or more droplets. Reprinted from [91,92] with permission from Springer; (**c**) and (**d**) demonstrate last moment of droplet merging, called decompression merging. Reprinted from [93] with permission from the American Physical Society (APS). Note: the arrows indicate the traveling direction of the droplets.

One widely used geometry for droplet merging consists of a widening channel follow by a narrower channel (Figure 11) [90–93]. In this geometry the droplet velocity decreases in the widening channel because of drainage of the continuous phase, after which it increases again upon entry in the narrow channel. Due to this changing flow field, two subsequent droplets are allowed to come close together and let the liquid that separates them drain away. Bremond *et al.* observed that the merging does not occur during the first interdroplet encounter in the extended channel, but rather during the separation stage of two droplets when the first droplet begins to enter the narrow channel (Figure 11c) [93]. The separation induces the formation of two facing protrusions (Figure 11d) which then bring the two interfaces close enough until they merge. Later Lai *et al.* reported a theoretical study based on this observation [111]. The created protrusions lead to a rapid increase of the surface area locally, and thus to destabilize the interface at certain locations. The conditions under which droplet merging occurs, can be predicted on the basis of their model. Alternatively in other channel geometries, droplets are merged by slowing down or stopping the leading droplet at a constriction [97,98], or in a channel with an array of pillar elements [94,95].

It should be noted that typically no surfactant is used in these passive merging experiments. However, the absence of surfactant has its drawbacks: unintended merging events can occur, and also the possibilities for further manipulation of the droplets after the merging can be limited. By exception, a case of passive merging of surfactant-covered droplets has also been reported. Mazutis *et. al.* demonstrated a channel design for merging droplets with significant asymmetry in size, both formed in the presence of surfactant [100]. However, undesired coalescence still occurred often. It is therefore often preferred to use surfactant stabilized drops and achieve merging with the help of external forces.

### 4.2. Active Merging

To achieve active and selective droplet merging, the most widely utilized method is to ensure the presence of an electric field at the location where two droplets meet. Link *et al.* showed that droplets can be merged by applying voltages with opposite sign across the two droplets during their formation. This is supposed to result in oppositely charged surfaces, which will attract each other strongly as soon as the droplets reach close proximity [72]. Alternatively, Chabert *et al.* achieved merging of individual droplet pairs via electrocoalescence (EC) [112]. This appears to be a promising method, although the mechanistic aspects of EC still remain to be understood [105,109,113,114].

**Figure 12.**
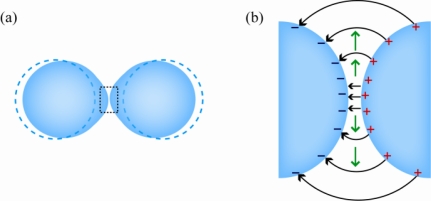
Two approaching droplets in an electric field. (**a**) Deformation from a sphere to a prolate spheroid occurs due to the electrical stress; (**b**) Close up showing local surface charges of opposite sign. Black arrows indicate the electric field. Green arrows indicate how ambient oil is squeezed out.

The general picture of EC is sketched in Figure 12. Due to the electric field, the two droplets experience an electrical (Maxwell) stress *σ*_E_ that tends to deform their shape from spherical to prolate spheroid. This stress is then balanced by the interface tension and the viscous stresses due to the deformation rate [115]. For Newtonian fluids at low Reynolds and Bond numbers, this is described by:

(2) $$\mu \nabla^{2}U\;-\;\nabla P\;=\;\nabla T$$

with *μ* the viscosity, *U* the velocity, *P* the pressure and *T* the stress field in each phase of the two-phase fluid. Since the velocity is continuous across the interface, the total stress difference (electric plus viscous) between inside and outside the droplet is balanced by the interfacial tension:

(3) $$nT\;=\;nT^{N}\;+\;nT^{E}\;=\;\sigma n\nabla_{S}n$$

where *n* is the unit normal vector at the interface, *σ* is the interfacial tension, ∇*_S_n* is the mean curvature of the interface, *T^E^* is the Maxwell stress tensor (proportional to the square of the applied electric field) and *T^N^* is the tensor of viscous forces [115]. Hence the field, the viscosities and the interfacial tension all play a role.

**Figure 13.**
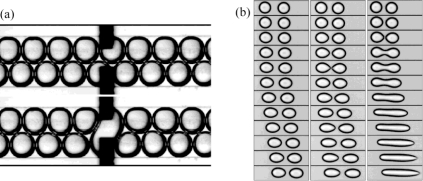
(**a**) Electrocoalescence of droplet pairs (electrodes are visible as shown black rectangles). Reprinted from [105] with permission from the American Institute of Physics (AIP); (**b**) EC as a function of interdroplet separation. Time sequences showing three different regimes: stable, partial merging and coalescence. Reprinted from [109] with permission from the American Physical Society (APS).

Priest *et al.* argued that EC involves an electric-field-induced dynamic instability of the oil/water interface, which subsequently leads to the formation of a liquid bridge and coalescence (Figure 13a) [105]. Thiam *et al.* analyzed the merging of droplets as a function of their separation distance (Figure 13b) [109], and also explained their observations in terms of a competition between electrical stress and restoring capillary pressure. Qualitatively speaking, it is clear that the electric field near the droplet surfaces can be amplified by dipole-dipole interactions between the droplets, and hence become stronger as the droplets get closer. It is conceivable that this will lead to destabilization of the surfaces [116]. Furthermore, also the surfactant molecules can be involved. In the case of surfactants with dipolar head-groups, a redistribution or re-alignment along the electric field lines can take place. Also this can destabilize the interface and lead to coalescence [117].

One of the first applications of EC in two-phase flow microfluidics was presented by Tan *et al*. [118]. Two droplets containing biological molecules were brought into an expanded channel and merged there, due to an electric field generated by an embedded electrode. Later, several variations based on this geometry were adopted to implement EC in microfluidic chips [72,109,112]. For each of these EC-based systems, droplet synchronization and precise electrode alignment are required.

**Figure 14.**
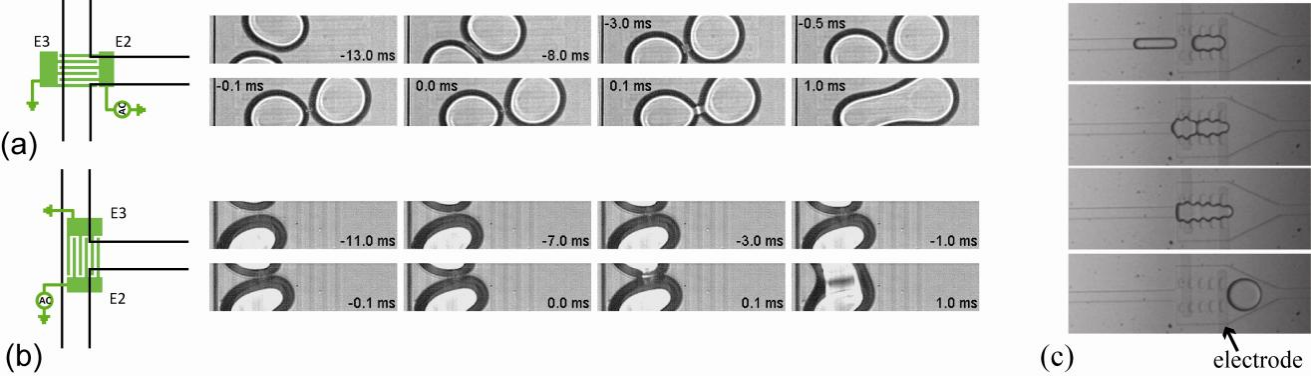
(**a**) and (**b**) Droplet merging on different orientations of the interdigitated electrodes. Reprinted from [119] with permission from the American Institute of Physics (AIP). (**c**) Droplet are stopped by pillar array and merged by EC. Reprinted from [110] with permission from American Chemical Society (ACS).

To overcome these limitations, Gu *et al.* used EW-induced *on demand* formation to obtain synchronization of two streams of produced droplets [119]. These two streams then meet at a T-junction where interdigitated electrodes are embedded (Figure 14a and b). Merging on demand can be achieved there based on EC. As illustrated in Figure 14c, Niu *et al.* depicted an alternative method, by combining a passive merging approach (a pillar array in the channel) with an active merging approach (built-in electrodes) [110]. In this scheme, the pillar array slows down and traps the droplet during the drainage of the oil phase. EC then occurs when droplets have reached close proximity. Also a double T-junction geometry with embedded electrodes has been reported in the context of active merging. In the system of Wang *et. al*., two series of droplets can be produced and merged at the same time [108].

Yet another method for the active merging of droplets is dielectrophoresis (DEP). A drawback of this method is that it requires rather high voltages, up to several kV [120–122]. Finally, thermo-capillary effects can also be cited as a mechanism to perform active merging of droplets [76,104,123,124]. Heating two adjacent droplets with a focused laser beam was reported to cause convective motions in the droplets, as well as depletion of surfactant molecules from the interface. Also this turned out to be effective for droplet merging.

## 5. Microchannel Fabrication

Microfabrication methods, which include film deposition, photolithography, isotropic or anisotropic etching steps and anionic bonding of a microchip, were initially achieved with silicon (Si) [125]. Later on glass substrates have also been used, while relying on similar fabrication procedures [126]. Nowadays the most commonly used microfluidic chips are made of polymers, in particular poly-dimethylsiloxane (PDMS), owing to its low expense and rapid prototyping [127,128]. The procedure for fabricating such microchips is based on soft lithography, involving photolithography steps for producing a mold, followed by casting of PDMS replicas from this mold, then bonding the PDMS slab to a glass slide to seal the microchannels [127,129]. Whereas initially molds were made from Si, later the “negative photoresist” SU-8 became more popular due to its simplicity of production. SU-8 in fact stands for a series of commercial resists, each having a different viscosity. This allows to achieve a wide range of channel heights, from a few microns to several hundred microns in one step (more details in [130]).

In the case of droplet formation in a microchip, controlling the wetting properties of the channel walls is essential. For instance, producing water droplets in oil phase requires hydrophobic channels, whereas producing oil droplets in water phase needs hydrophilic channels. Some strategies can be used to alter the wettability of the channel walls with regard to the required droplet formation. For glass or Si-based microchips, treatments such as silanization and siliconization, can be implemented to modify the hydrophilic surface into hydrophobic [47,131,132]. Oxygen plasma treatment can alter the naturally hydrophobic PDMS surface into hydrophilic temporarily [127]. If this is not sufficient to achieve the desired wetting properties, a permanent surface modification, such as acrylic acid polymer grafting [133] or sol-gel coating [134] can be used to make PDMS surface hydrophilic. Another motivation for surface modification of PDMS lies in its poor chemical compatibility, causing swelling and deformation in the presence of strong organic solvents. Polymer grafting methods, using acrylic acid or poly(ethylene glycol) (PEG), are widely used to counteract this effect [133,135].

Achieving a reliable surface modification of PDMS is still a challenging task. An interesting alternative class of polymers is fluoropolymers, which exhibit excellent chemical compatibility, in addition to similar properties of PDMS (transparency, flexibility and conformability) [136,137]. However, the bonding of fluoropolymer and substrate is quite weak, which limits applications under high working pressures. Also this kind of device cannot be used at high temperature [138].

**Figure 15.**
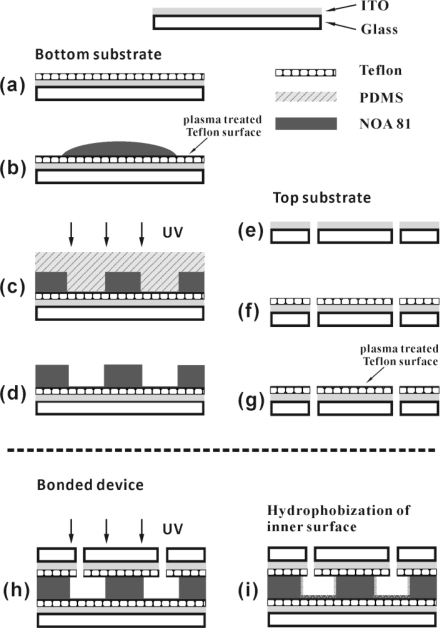
Fabrication process of NOA 81-based microfluidic device. (**a**)–(**d**) Imprinting NOA 81 as channel structure on a Teflon AF-coated ITO glass slide; (**e**)–(**g**) Coating Teflon AF on the top substrate with drilled holes for inlet and outlet connections; (**h**) Bonding both bottom and top parts under UV exposure; (**i**) Silanization treatment to modify inner surface hydrophobic. Reprinted from [69] with permission from Royal Society of Chemistry (RSC).

Another issue of PDMS is its low elastic modulus, which limits the production of microchannels with low aspect ratio or very small dimensions. Currently there is much attention on developing a rapid prototyping technique using ultraviolet (UV) curable polymers [69,139–144]. Gu *et al.* demonstrated the fabrication of a hybrid microchip based on the UV curable material of Norland Optical Adhesive (NOA), followed by a silanization treatment of inner surface. The fabrication process is demonstrated in Figure 15. The channel structure is made of NOA 81; the bottom and top substrates are Teflon AF-coated ITO glass slides. NOA 81 exhibits excellent adhesion on different materials, such as glass, metal, even Teflon AF with known strong chemical inertness. Such NOA-based microchips reveal greater chemical compatibility and higher elastic modulus than PDMS. Thus NOA-based microchips can be used in various organic solvent environments. It also presents alternative way to implement electric components into microfluidic device.

Hot embossing technique is an alternative soft lithographic method for fabrication of microchips using thermoplastic polymers. The number of available thermoplastic materials has strongly increased in the last few years [145–148]. Tsao *et al.* reviewed the bonding of thermoplastic polymer to substrates [149]. Moreover, this class of polymers is suitable for device fabrication on a large scale.

## 6. Conclusions

In conclusion, we have reviewed recent progress in the field of droplet-based two-phase flow microfluidics regarding two fundamental droplet manipulation processes: formation and merging. The formation and merging of droplets, being the two key steps of many operations, have been optimized for a large variety of applications, ranging from emulsification at controlled droplet size to the use of droplets as microreactors. Besides purely hydrodynamic manipulation electric signals transmitted via microelectrodes are also increasingly used to enhance control. With the latter, the formation and merging of droplets can be achieved on-demand and with high precision. Thus the hybrid microchips (microfluidic channel combined with extra components, for instance electrodes) have attracted many researchers to study and this trend is anticipated to continue in the years to come. In particular, the use of electrowetting in combination with patterned electrodes embedded into the channel walls is expected to enhance the flexibility of multifunctional microfluidic devices substantially. Also innovative microfabrication technologies have undergone rapid development to optimize droplet manipulations in the last few years.
